# Homocysteine levels in schizophrenia and affective disorders—focus on cognition

**DOI:** 10.3389/fnbeh.2014.00343

**Published:** 2014-10-06

**Authors:** Ahmed A. Moustafa, Doaa H. Hewedi, Abeer M. Eissa, Dorota Frydecka, Błażej Misiak

**Affiliations:** ^1^School of Social Sciences and Psychology and Marcs Institute for Brain and Behaviour, University of Western SydneySydney, NSW, Australia; ^2^Psychogeriatric Research Center, Department of Psychiatry, School of Medicine, Ain Shams UniversityCairo, Egypt; ^3^Department and Clinic of Psychiatry, Wroclaw Medical UniversityWroclaw, Poland; ^4^Department of Genetics, Wroclaw Medical UniversityWroclaw, Poland

**Keywords:** homocysteine, depression, bipolar disorder, schizophrenia, hyperhomocysteinemia, cognition, brain substrates

## Abstract

Although homocysteine (Hcy) has been widely implicated in the etiology of various physical health impairments, especially cardiovascular diseases, overwhelming evidence indicates that Hcy is also involved in the pathophysiology of schizophrenia and affective disorders. There are several mechanisms linking Hcy to biological underpinnings of psychiatric disorders. It has been found that Hcy interacts with NMDA receptors, initiates oxidative stress, induces apoptosis, triggers mitochondrial dysfunction and leads to vascular damage. Elevated Hcy levels might also contribute to cognitive impairment that is widely observed among patients with affective disorders and schizophrenia. Supplementation of vitamins B and folic acid has been proved to be effective in lowering Hcy levels. There are also studies showing that this supplementation strategy might be beneficial for schizophrenia patients with respect to alleviating negative symptoms. However, there are no studies addressing the influence of add-on therapies with folate and vitamins B on cognitive performance of patients with schizophrenia and affective disorders. In this article, we provide an overview of Hcy metabolism in psychiatric disorders focusing on cognitive correlates and indicating future directions and perspectives.

## Introduction

Homocysteine (Hcy) is one of the non-protein amino acids that is produced in one-carbon metabolism. Two enzymatic pathways are involved in Hcy metabolism—re-mehtylation to methionine and trans-sulfuration to cysteine and taurine. The efficiency of Hcy catabolism depends on the availability of folate, vitamin B12 and vitamin B6. Tans-sulfuration to cysteine, which forms glutathione, is catalyzed by cystathionine beta synthase (CBS) and cystathionase. In turn, conversion from Hcy to methionine is a multistep reaction with a number of enzymes being involved including Hcy methyltransferase, methionine synthase (MS) and methionine synthase reductase (MTRR), as well as the methylenetetrahydrofolate reductase (MTHFR; Scott and Weir, 1998). There are two common polymorphisms located in the *MTHFR* gene—C677T and A1298C that may lower the activity of MTHFR and lead to increased Hcy levels. The most common one—C677T polymorphism, which is present in 10–12% of population (Gilbody et al., 2007), contributes to the expression of a thermolabile variant of MTHFR. Other factors might also increase Hcy level including higher age, male gender, cigarette smoking, alcohol abuse or dependence, low dietary intake of folate and vitamins B, renal dysfunction and certain medications (e.g., sodium valproate and lamotrigine, diuretics, fibrates) (Frankenburg, 2007). In addition, there is an inverse relationship between Hcy and both folate and vitamin B12 levels (Yoshino et al., 2010).

Several lines of evidence indicate that Hcy serves as an important atherosclerotic factor. It has been found that Hcy may induce vascular damage via initiating oxidative stress and reducing the availability of nitric oxide that is a powerful vasodilator (Perna et al., 2003). These mechanisms underlie well-established links between elevated Hcy levels or *MTHFR* polymorphisms and cardiovascular diseases including coronary artery disease, myocardial infarction, cerebrovascular disease and peripheral occlusive disease (Mangoni and Jackson, 2002; Trimmer, 2013).

In the recent years, there is a growing interest in the causative links between Hcy and neuropsychiatric disorders. High Hcy levels are increasingly recognized as a risk factor for age-related cognitive deficits together with various types of dementia (Stanger et al., 2009). Studies in this field have provided several links between Hcy and domains of cognitive functioning (Faux et al., 2011; Kim et al., 2013). However, less attention has been paid to cognitive correlates of elevated Hcy level in psychiatric disorders including schizophrenia and affective disorders. In this article, we review the role of Hcy in the pathophysiology of psychiatric disorders including schizophrenia and affective disorders focusing on cognitive correlates.

## Mechanisms of homocysteine action—the relevance to psychiatric disorders

The exact neural and behavioral mechanism of Hcy action is not known. It seems that the interaction of Hcy with glutamatergic transmission is the most relevant mechanism explaining the association between Hcy and schizophrenia or affective disorders. Both Hcy and its oxidative metabolite—homocysteic acid—serve as agonists within NMDA receptors (Klancnik et al., 1992; Zhang and Lipton, 1992; Lipton et al., 1997). Stimulation of NMDA receptors by Hcy increases calcium influx that exerts neurotoxic effects (Ho et al., 2002). However, in the presence of low concentrations of glycine, Hcy acts as a partial antagonist within the glycine site of NMDA receptors. Thus, in case of low glycine level Hcy manifests its neuroprotective activity (Lipton et al., 1997) and only high Hcy concentrations may be toxic. On the other hand, when glycine levels are high (after head trauma or stroke), low Hcy levels become toxic (Alam et al., 1998). This dual action of Hcy within NMDA receptors may explain why elevated Hcy levels might be implicated in schizophrenia, in which hypofunction of glutamatergic transmission has been reported and depression that is characterized by up-regulated glutamatergic activity.

Also, various studies have suggested that Hcy might regulate the function of other neuromodulators, such as acetylcholine (Chen et al., 2011) and dopamine, and serotonin (Gao et al., 2011). Specifically, Gao et al. (2011) have reported that rats with hyperhomocysteinemia have lower levels of dopamine and serotonin in the cortex than control rats. Other studies suggest that Hcy regulates synaptic plasticity in the hippocampus (Christie et al., 2005; Algaidi et al., 2006). These prior studies suggest that Hcy has multiple functions in the brain; this can likely explain its links to various psychiatric disorders, including schizophrenia and affective disorders.

**Figure 1:**
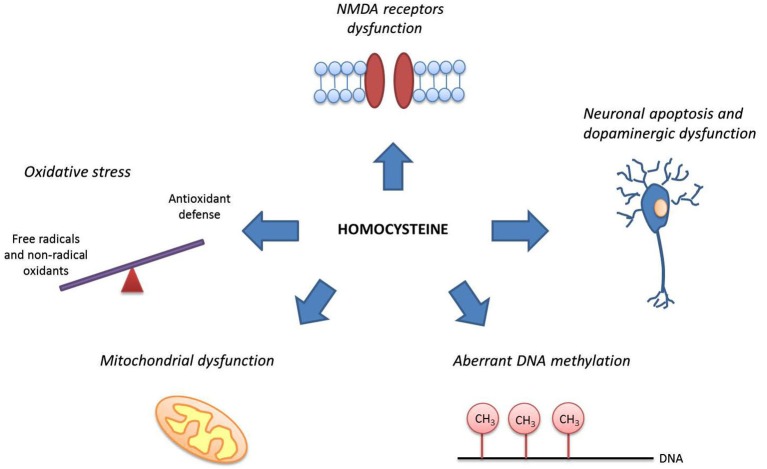
**Mechanisms of homocysteine action as relevant to neurological and psychiatric disorders**. Homocysteine may interact with NMDA receptors altering glutamatergic transmission, exert toxic effects on dopaminergic neurons, initiate neuronal apoptosis, induce oxidative stress, lead to mitochondrial dysfunction and influence DNA methylation altering gene expression.

Animals exposed to Hcy exhibit compromised brain energy metabolism (Streck et al., 2003), altered long-term potentiation, disturbances of synaptic plasticity and cognitive impairment in terms of spatial learning (Algaidi et al., 2006) and memory deficits (Streck et al., 2004). Heterozygous and homozygous *Mthfr* knockout mice are also characterized by neurodevelopmental retardation and altered cerebellar morphology (Chen et al., 2001). Other mechanisms of Hcy toxicity that might be relevant to the pathophysiology of schizophrenia and affective disorders include oxidative stress (Koz et al., 2010; Loureiro et al., 2010; Dietrich-Muszalska et al., 2012), neuronal apoptosis (Wang et al., 2012), vascular damage (Brown et al., 2007) and aberrant DNA methylation (Bromberg et al., 2008, 2009; Kinoshita et al., 2013; Figure 1). Neural studies have shown that Hcy acts on various brain regions, including the hippocampus (den Heijer et al., 2003; Matté et al., 2009; Chen et al., 2011), cortex (den Heijer et al., 2003), and the basal ganglia (Genedani et al., 2010). Higher Hcy levels lead to atrophy in the frontal, parietal, and temporal areas (Rajagopalan et al., 2011).

## Homocysteine and cognition in healthy individuals

Homocysteine plays an important role in behavioral and cognitive processes as shown in studies measuring Hcy levels in healthy elderly subjects (Prins et al., 2002; Dufouil et al., 2003; Teunissen et al., 2003; Nurk et al., 2005; Feng et al., 2006; Hooshmand et al., 2012). For example, van den Kommer et al. (2010) reported that higher Hcy levels are associated with slow information processing speed in healthy participants. Further, Nurk et al. (2005) found that impaired episodic memory performance is associated with increased Hcy levels in healthy individuals. Along the same lines, Garcia et al. (2004) revealed that impaired performance in the Stroop test correlates with higher levels of Hcy. Studies on the role of Hcy in cognitive performance in healthy subjects have shown that Hcy is specifically involved in episodic memory (Faux et al., 2011; Narayan et al., 2011), spatial learning (Pirchl et al., 2010), reversal learning (Christie et al., 2005; Algaidi et al., 2006), and executive function (Narayan et al., 2011). However, it is debatable whether Hcy plays a role in working memory processes, as some studies have found they are not related (Narayan et al., 2011), while other studies found that lowering Hcy levels enhances working memory (Macpherson et al., 2012).

Recently published results reveal associations between total Hcy levels and cognitive functions in healthy subjects. It has been found that lower overall cognitive performance measured by Cambridge Cognitive Examination (CAMCOG) are associated with higher Hcy levels (Budge et al., 2002). This study also revealed an inverse correlation between hippocampal volume and Hcy levels (Budge et al., 2002). Other studies have found a positive correlation between total Hcy levels and ventricle-brain ratios in the anterior and middle ventricular regions in elderly participants (Sachdev et al., 2002). It has also been reported that higher Hcy levels are associated with lower scores in Mini Mental State Examination (MMSE; Kalmijn et al., 1999). It has been demonstrated that impaired cognition in elderly participants correlates with Hcy levels, especially for psychomotor speed and memory functions (Prins et al., 2002).

Recent data show that higher Hcy levels are associated with silent brain infarctions and subcortical white matter lesions in older adults (Vermeer et al., 2002). Higher Hcy levels have been associated with increased prevalence of silent brain infarction and decreased brain volume in comparison with subjects having lower total Hcy (Morris, 2003).

## Homocysteine in psychiatric disorders

Total Hcy level changes have also been shown to be associated with many psychiatric disorders, including schizophrenia and affective disorders. These observations stimulated further studies on the association between elevated Hcy levels and neuropsychiatric symptoms and disorders.

Patients having cognitive disorders and depression have been reported in many studies to have low vitamin B12 and folate levels. In 1980, an important finding by Shorvon et al. (1980) was published on the neuropsychiatric manifestations in megaloblastic anemia that occurred due to low folate or vitamin B12 levels. Their study revealed that up to 56% of patients with affective disorders have serum folate deficiency (Shorvon et al., 1980). Below, we describe the relationship between changes in Hcy levels and schizophrenia, depression, and bipolar disorder.

### Schizophrenia

In 1975, Freeman et al. (1975) described a case of homocystinuria, caused by a deficient MTHFR activity, accompanied by psychotic-like behavior that responded to folate treatment. More recently, a new hypothesis for the development of schizophrenia has been proposed—the DNA polymorphism-diet-cofactor-development (DDCD) hypothesis (Johnson, 1999). This hypothesis states that mutations of genes related to folate and vitamins B metabolism potentiated by maternal dietary vitamin deficiencies contribute to the development of schizophrenia. Total Hcy serum levels in schizophrenia were first measured by Regland et al. (1995). In this study, elevated Hcy levels were found in 9 out of 20 schizophrenic patients (Regland et al., 1995).

Subsequently, elevated total Hcy levels have been widely described in various subgroups of schizophrenia patients (Muntjewerff et al., 2006; Nishi et al., 2014) including drug-naïve first-episode psychosis subjects (Kale et al., 2010; Ayesa-Arriola et al., 2012; García-Bueno et al., 2013) and chronic schizophrenia patients (Eren et al., 2010). Total Hcy level has been found to negatively correlate with folate and vitamin B12 levels in this group of patients (Bouaziz et al., 2010). In addition, some authors have found that Hcy levels are higher especially in young male schizophrenia patients (Levine et al., 2002). It has also been estimated that a 5-μmol increase in plasma Hcy level may increase the risk of schizophrenia by 70% (Muntjewerff et al., 2006). Several studies have proved a positive correlation between Hcy levels and the severity of schizophrenia negative symptoms (Goff et al., 2004; Petronijević et al., 2008; Bouaziz et al., 2010; Misiak et al., 2014). These studies are in concordance with the studies showing a negative correlation between duration of untreated psychosis (DUP) and Hcy levels (Ayesa-Arriola et al., 2012; Misiak et al., 2014). The association of increased Hcy levels with schizophrenia psychopathology has provided grounds for add-on therapies with vitamin supplementation (Hill et al., 2011; Roffman et al., 2013). The largest randomized, double-blind and placebo-controlled study of folic acid and vitamin B12 supplementation revealed the improvement of negative symptoms in schizophrenia patients. However, this supplementation strategy was effective only in patients being homozygotes of the 484T > C polymorphism in the *FOLH1* gene that encodes folate hydrolase involved in intestinal folate transport (Roffman et al., 2013).

Elevated Hcy levels found in first-episode psychosis patients suggest that one-carbon metabolism alterations may share common genetic underpinnings with schizophrenia. Another proof for this assumption is that siblings of schizophrenia patients are also characterized by increased plasma Hcy levels (Geller et al., 2013) and schizophrenia patients with positive family history of schizophrenia in first or second degree relatives have significantly higher Hcy levels compared to those with negative family history of schizophrenia (Misiak et al., 2014). Several studies have reported an association of two common polymorphisms in the *MTHFR* gene (C677T and A1298C) with schizophrenia (Lewis et al., 2005; Muntjewerff et al., 2006; Gilbody et al., 2007; Shi et al., 2008). Furthermore, these polymorphisms have been found to predict the development of metabolic syndrome following the treatment with antipsychotics or at least might be associated with increased incidence of metabolic disturbances, such as visceral obesity, impaired metabolism of glucose and lipids (Misiak et al., 2013). Furthermore, schizophrenia patients with the comorbid metabolic syndrome are characterized by higher Hcy levels in comparison with those, who do not meet the criteria of metabolic syndrome (Vuksan-Ćusa et al., 2011, 2013).

Although the *MTHFR* gene polymorphisms are known to influence the risk of metabolic adverse effects of antipsychotics, the influence of antipsychotic treatment on Hcy requires further investigation due to scarcity of well-designed studies. There is only one observational study on drug-naïve first episode schizophrenia patients showing the lack of significant changes in Hcy levels in the course of antipsychotic pharmacotherapy (Bicikova et al., 2011). Another study on acutely relapsed schizophrenia patients has revealed significantly higher Hcy levels during symptomatic exacerbation than during the remission phase (Petronijević et al., 2008). In turn, the cross-sectional study by Eren et al. (2010) on chronic schizophrenia patients revealed significantly lower levels of plasma folate, but not Hcy or vitamin B12, in patients receiving higher doses of typical antipsychotics (chlorpromazine equivalent >400 mg). Another cross-sectional study revealed no significant difference in Hcy level between schizophrenia patients receiving clozapine in monotherapy and healthy controls (Wysokiński and Kłoszewska, 2013). There is also one study showing a positive relationship between Hcy levels and N-desmethyl-olanzapine concentration that is one of the main olanzapine metabolites (Lu et al., 2013). These inconsistent results might be attributed to heterogenous methodology such as the recruitment of different patients defined by illness duration or symptomatic presentation, as well as the lack of adjustment for possible confounders including the *MTHFR* genotype, dietary habits, cigarette smoking or other known factors influencing Hcy metabolism.

Several studies have established direct links between the *MTHFR* gene polymorphisms and cognitive dysfunction in terms of executive function and blunted response to errors in schizophrenia. It has been found that the 677T variant of the *MTHFR* gene induces a dose-dependent blunting of dorsal anterior cingulate cortex activation in response to errors using the antisaccade paradigm (Roffman et al., 2011b), positively correlates with impairments of verbal fluency (Roffman et al., 2007) and interacts with the 108Val allele in the *COMT* gene increasing the number of perseverative errors on the Wisconsin Card Sorting Task (WCST; Roffman et al., 2008b). Although the *MTHFR* gene variants have been reported to influence certain domains of cognitive functioning in schizophrenia patients, Hcy levels have not been found to correlate with cognitive impairment in first-episode schizophrenia spectrum disorders patients (Ayesa-Arriola et al., 2012). These discrepancies suggest that other Hcy-independent consequences of one-carbon metabolism dysfunction due to genetic factors are implicated in the occurrence of cognitive impairment in schizophrenia. Given that the 677T allele in the *MTHFR* gene is associated with lower genomic DNA methylation (Friso et al., 2002), it might be hypothesized that epigenetic phenomena are involved in cognitive impairment in schizophrenia. Furthermore, the 677T variant enhances dopamine metabolism (Roffman et al., 2008a,b), which is linked to schizophrenia pathophysiology and is implicated in the activation of dorsal anterior cingulate cortex in response to errors (Holroyd and Coles, 2002) and influences prefrontally-mediated executive functioning (Tan et al., 2007).

### Depression

Several studies have established that depressive episodes may predict the development of cardiovascular diseases (de Jonge et al., 2014). These findings suggest that depression is linked to co-occurring metabolic deregulation increasing cardiovascular risk. Indeed, elevated Hcy levels have been shown in major depression (Folstein et al., 2007; Yapislar et al., 2012; Delport et al., 2014; Lok et al., 2014). Notably, it has been found that Hcy level negatively correlates with vitamin B12 and folate levels in depressed patients (Ebesunun et al., 2012). There are also studies showing that the *MTHFR* C677T polymorphism may increase the susceptibility to major depression (Wu et al., 2013; Delport et al., 2014; Lok et al., 2014; Shen et al., 2014). Interestingly, it has been found that the *MTHFR* 677T allele may interact with childhood traumatic events influencing the time to recurrence in major depressive disorder (Lok et al., 2013). Indeed, the carriers of the *MTHFR* 677T allele with childhood traumatic events had shorter time to recurrence of major depressive disorder in comparison with those without such events. These findings corroborate emerging evidence indicating that posttraumatic stress disorder (PTSD) patients are also characterized by elevated Hcy levels (Levine et al., 2008; Jendricko et al., 2009).

In the recent study with older adults, it was found that serum folate levels correlate with the severity of depressive symptoms (Ebly et al., 1998). In studies that failed to prove an association between low serum folic acid and depression, there was a negative correlation between folate level and the duration of the depressive episode, or a negative correlation between folate level and length of hospitalization and therefore with treatment outcome. Regarding the severity of depression, patients with lower folate levels were more severely depressed than those with normal folate levels (Alpert et al., 2000). In the Womens Health and Aging Study, low vitamin B12 levels were reported in elderly disabled community participants and significant vitamin B12 deficiency was more common among depressed than healthy participants. Significant vitamin B12 deficiency was associated with a two-fold higher risk of developing severe depression (Penninx et al., 2000). Interestingly, in the study by Gabryelewicz et al. (2007), depression and higher baseline Hcy levels were the strongest predictors of conversion from mild cognitive impairment (MCI) to dementia. Elevated total Hcy levels were also observed in the study of 213 patients with major depression compared with controls (Fava et al., 1997). S-Adenosyl Methionine (SAM), a precursor of Hcy, is used in some countries as an effective adjuvant therapy in the treatment of depression. On the basis of a meta-analysis, Bressa (1994) also suggested that SAM can act as an antidepressive agent. S-Adenosyl Methionine has also been found to be effective in the treatment of depression related to Parkinsons disease (Di Rocco et al., 2000). Studies on patients with geriatric depression have revealed correlations between Hcy and cognitive performance. In this group of patients, Hcy level positively correlated with language processing and processing speed (Alexopoulos et al., 2010).

### Bipolar disorder

Although elevated Hcy levels have been repeatedly reported in bipolar disorder patients (Baek et al., 2013), no significant differences have been found across various mood states (Chiarani et al., 2013). Studies on bipolar disorder indicate that high Hcy levels are significantly more frequent among males than females with bipolar depressive episode (Permoda-Osip et al., 2013b, 2014b). Similarly to schizophrenia and major depression patients, an inverse relationship between Hcy and both folate vitamin B12 levels has been demonstrated in bipolar disorder subjects (Permoda-Osip et al., 2013b). However, it has been found that Hcy level negatively correlates with the level of endothelial damage markers including E-selectin and intracellular adhesion molecule-1 (ICAM-1) in bipolar depression subjects suggesting that the pathways of cardiovascular risk are not associated with Hcy metabolism in this group of patients (Permoda-Osip et al., 2013b).

As similar to schizophrenia, two common polymorphisms in the *MTHFR* gene (C677T and A1298C) might increase the risk of bipolar disorder and predict the development of comorbid metabolic syndrome suggesting the existence of common genetic underpinnings (Peerbooms et al., 2011; Ellingrod et al., 2012). There is also one study showing an association between the T833C polymorphism in the *CBS* gene and bipolar disorder risk (Permoda-Osip et al., 2014a).

However, in contrast to studies on schizophrenia, evidence for the influence of Hcy on cognition is more convincing. There are studies showing an inverse relationship between plasma Hcy and verbal learning, executive function or immediate memory in euthymic bipolar disorder patients (Dittmann et al., 2007, 2008; Osher et al., 2008). It should be noted that two studies consistently reported the correlation between Hcy levels and executive functioning measured in terms of cognitive flexibility tapped by Trail Making Test subtest B (Osher et al., 2008) and perseverative errors assessed on WCST (Dittmann et al., 2007). Notably, these findings overlap with the influence of *MTHFR* polymorphisms on cognitive performance reported in schizophrenia patients (Roffman et al., 2007, 2008a,b, 2011a,b). As mentioned above, the C677T polymorphism in the *MTHFR* gene has been associated with greater deficits of executive functioning assessed on WCST in schizophrenia subjects (Roffman et al., 2007).

Cognitive deficits due to elevated Hcy level might be particularly prominent among older bipolar disorder patients or those with a delayed onset of the disorder (Dias et al., 2009). However, it should be kept in mind that aging increases Hcy levels and some cognitive deficits due to hyperhomocysteinemia may occur regardless of depression. It has been shown that hyperhomocysteinemia worsens cognitive performance in tests of immediate or delayed memory, as well as global cognitive functioning in older subjects (Ford et al., 2013). It should also be noted that patients with bipolar disorder might exhibit higher Hcy levels due to the treatment with mood stabilizers. Indeed, experimental studies have revealed that sodium valproate inhibits methionine adenosyltransferase, while lamotrigine serves as a weak dihydrofolate reductase inhibitor leading to lower functional folate levels despite of normal blood levels of folate (Baek et al., 2013).

There are two randomized placebo controlled trials investigating the efficacy of folic acid supplementation in bipolar depression. These studies revealed that folic acid may enhance lithium prophylaxis (Coppen et al., 1986) and antidepressant action of fluoxetine in females (Coppen and Bailey, 2000). Furthermore, it has been found that the augmentation of sodium valproate with folic acid might be beneficial in terms of reducing manic symptoms (Behzadi et al., 2009). Inconsistent results also indicate that higher vitamin B12 level may predict favorable response to single ketamine infusion in bipolar depression patients (Permoda-Osip et al., 2013a; Lundin et al., 2014). Ketamine is an NMDA receptor antagonist, emerging as a therapeutic strategy in treatment-resistant depression (Naughton et al., 2014).

## Future directions and conclusion

Undoubtedly, elevated Hcy levels are associated with a wide spectrum of psychiatric disorders including particularly schizophrenia and affective disorders. It might be assumed that the dual action of Hcy (as agonist or antagonist) within NMDA receptors (Lipton et al., 1997) explains why elevated Hcy levels are involved in the pathophysiology of both schizophrenia and affective disorders. This association is probably strengthened by high prevalence of metabolic syndrome and its single components, which is a consequence of antipsychotic treatment. Emerging evidence indicates that high Hcy levels may, to some extent, account for cognitive deficits among these groups of patients. It seems that the influence of Hcy on executive functioning occurs regardless of a psychiatric diagnosis since this correlation has been found both in schizophrenia and bipolar disorder patients. In this regard, it is also recommended to investigate the influence of Hcy on cognition in healthy adults in order to determine the extent of cognitive deficits that are the consequence of elevated Hcy levels. Further, future studies should investigate the relationship between Hcy levels in these patient populations on and off their medications to tease apart the relationship between Hcy, psychiatric disorders, and treatment duration or type of medications.

There is still a scarcity of studies investigating the relationship between Hcy and cognitive deficits in drug-naïve first-episode patients and high-risk populations. These studies are warranted as they may indicate the correlation between Hcy levels and early cognitive deficits that are strictly associated with schizophrenia and affective disorders regardless of medication and disease duration. Irrespective of a diagnostic subgroup, future studies should take into account the confounding effect of such variables as body weight, dietary habits, smoking or alcohol consumption that are less frequently controlled, as previous studies have shown that these variables are correlated with Hcy levels and may thus confound findings in the relationship between psychiatric disorders and Hcy levels. Given the largely known contribution of Hcy to the etiology of various types of dementia, it might be also beneficial to address the role of Hcy in the neuroprogression of cognitive deficits that is widely observed particularly in affective disorders and remains the matter of dispute in schizophrenia. Longitudinal measurements of Hcy along with assessment of cognitive functioning that take into account the effects of age as an confounding factor are required to initiate this vein of research.

It should be noted that supplementation of folic acid and vitamins B may normalize Hcy levels. However, we are not aware of any studies addressing the efficacy of supplementation strategies with respect to alleviating cognitive deficits among patients with schizophrenia or affective disorders. Similarly, a gap exists in addressing the influence of antipsychotic treatment on Hcy metabolism, and its correlations with cognitive processes, which should be the focus in future work. As mentioned above, there is only one cross-sectional study revealing a negative correlation between folate levels and high chlorpromazine equivalents (>400 mg/day) (Eren et al., 2010) in chronic schizophrenia patients and one observational study performed in a small sample of drug-naïve first-episode schizophrenia patients reporting no significant alterations in Hcy levels in the course of antipsychotic treatment (Bicikova et al., 2011). Another study revealed a decrease in Hcy levels during the treatment of acute relapse of schizophrenia (Petronijević et al., 2008). This issue is important due to the known influence of certain antipsychotics on the development of obesity and its metabolic consequences, such as dyslipidemia, diabetes or hypertension that have been found to influence cognitive performance in schizophrenia patients (Lancon et al., 2012; Lindenmayer et al., 2012; Boyer et al., 2013; Li et al., 2014).

Results of studies based on candidate gene approach and investigating genetic variation within the Hcy metabolism enzymes should be interpreted with caution. Previous genome-wide association studies (GWAS) have not confirmed the association between polymorphisms in the *MTHFR* gene or other genes implicated in Hcy metabolism and schizophrenia (Yoshimi et al., 2010; Lencz et al., 2013; Ripke et al., 2013, 2014; Ivorra et al., 2014; Saito et al., 2014) or bipolar disorder (Sklar et al., 2011; Kuo et al., 2014; Mühleisen et al., 2014; Xu et al., 2014) risk. There is only one genome-wide linkage analysis of recurrent depressive disorder providing evidence for linkage on chromosome region 1p36 including the *MTHFR* gene with the LOD score for female-female pairs estimated at 2.73 (McGuffin et al., 2005). In this regard and taking into account the involvement of Hcy pathway in several physical health impairments, it might be hypothesized that discordant results of GWAS and candidate gene approach studies may originate from genetic heterogeneity across studied populations and various clinical phenotypes including distinct somatic comorbidities that have also been attributed to polymorphisms in the *MTHFR* gene.

## Conflict of interest statement

The authors declare that the research was conducted in the absence of any commercial or financial relationships that could be construed as a potential conflict of interest.
