# Corrigendum: Homocysteine levels in schizophrenia and affective disorders—focus on cognition

**DOI:** 10.3389/fnbeh.2015.00081

**Published:** 2015-04-10

**Authors:** Ahmed A. Moustafa, Doaa H. Hewedi, Abeer M. Eissa, Dorota Frydecka, Błażej Misiak

**Affiliations:** ^1^School of Social Sciences and Psychology and Marcs Institute for Brain and Behaviour, University of Western SydneySydney, NSW, Australia; ^2^Psychogeriatric Research Center, Department of Psychiatry, School of Medicine, Ain Shams UniversityCairo, Egypt; ^3^Department and Clinic of Psychiatry, Wroclaw Medical UniversityWroclaw, Poland; ^4^Department of Genetics, Wroclaw Medical UniversityWroclaw, Poland

**Keywords:** homocysteine, depression, bipolar disorder, schizophrenia, hyperhomocysteinemia, cognition, brain substrates

The identification number of the project was incorrect. The article was supported by the project DEC-2011/03/N/NZ5/00248 funded by National Science Centre.

## Conflict of interest statement

The authors declare that the research was conducted in the absence of any commercial or financial relationships that could be construed as a potential conflict of interest.

